# On the identification of internal solitary waves from moored observations in the northern South China Sea

**DOI:** 10.1038/s41598-023-28565-5

**Published:** 2023-02-23

**Authors:** Qinglong Gong, Liang Chen, Yina Diao, Xuejun Xiong, Jilin Sun, Xianqing Lv

**Affiliations:** 1 Frontier Science Center for Deep Ocean Multispheres and Earth System (FDOMES) and Physical Oceanography Laboratory, Ocean University of China, Qingdao, 266100 China; 2 First Institute of Oceanography, Ministry of Natural Resources, Qingdao, 266061 China; 3 Founctional Laboratory for Regional Oceanography and Numerical Modeling, Pilot National Laboratory for Marine Science and Technology (Qingdao), Qingdao, 266237 China; 4 Laboratory for Regional Oceanography and Numerical Modeling, Pilot National Laboratory for Marine Science and Technology (Qingdao), Qingdao, 266237 China; 5 Qingdao National Laboratory for Marine Science and Technology, Qingdao, 266200 China

**Keywords:** Ocean sciences, Physical oceanography

## Abstract

Based on the temperature data recorded by a mooring system deployed in the northern South China Sea (SCS), this study compares the traditional methods of identifying and extracting internal solitary waves (ISWs): the whole water column isotherm (WCI) method and the single isotherm (SI) method, and proposes a novel method: the temperature superposition (TS) method. Results indicate that the TS method identifies the most ISWs, followed by the WCI method, and the SI method identifies the fewest. When the ISW amplitudes are smaller than 60 m, the TS method can identify the most ISWs. When the ISW amplitudes are greater than 60 m, the number of ISWs identified by the three methods is the same. The TS method removes the internal tides, takes into account the temperature of all depth layers, and amplifies the signal of ISWs to a certain extent, so as to effectively identify ISWs.

## Introduction

Internal solitary waves (ISWs) are waves with finite-amplitude, which owe their existence to a balance between nonlinear wave-steepening processes and linear wave dispersion^1^. ISWs with large amplitudes and strong current velocities are widely distributed around the continental shelf edges, steep subsurface slopes of islands and seamounts, and sills in straits, where strong barotropic tidal currents flow over steep topographies or interact with thermoclines^2–6^.

The South China Sea (SCS), as the largest marginal sea of the northwestern Pacific Ocean, has significant ISWs^7–14^. The main source of ISWs in the SCS is the Luzon Strait, where tidal currents interact with topography and generate internal tides in the form of wave beams^15–17^, which propagate eastward into the Pacific Ocean and westward into the SCS^18,19^. During the propagation process, the solitons emerge through nonlinear steepening, and the internal tides disintegrate into ISWs^20^. In addition, the nonlinear internal waves can also be excited near the Luzon Strait, such as lee waves^21^. When lee waves cross the submarine ridge, it will evolve into ISWs. The main propagation direction of ISWs in the SCS is northwest. When the topography and water depth change during the propagation process, the propagation direction, wave speed and even the polarity of ISWs change^22^. In addition, ISWs are affected by other ocean dynamical processes with different time and space scales, such as mesoscale eddies, large-scale circulation (the upstream SCS western boundary current) and tidal modulation^23–25^. Due to the retardation and acceleration effects on two sides of mesoscale eddies, the ISW front is distorted^23^. The upstream SCS western boundary current can make ISWs speed up while the ISW amplitudes reduce^24^. The tidal currents can modulate the stratification and induce sheared currents, which results in a cross-shelf movement of the critical point, where the polarity of ISWs change^25^. ISWs eventually shoal on the continental slope and shelf until they are dissipated^26^. The typical time scale, horizontal scale and amplitude of ISWs are tens of minutes, 1 km and over 200 m, respectively^27–29^. In terms of the wave modes, the first baroclinic mode (mode-1) and the second baroclinic mode (mode-2) ISWs are dominant in the northern SCS^30,31^. The mode-1 ISW only shows the downward or upward displacement of isotherms in the whole water column, while the mode-2 ISW usually shows both upward and downward displacements of isotherms in the water column^32^.

Many studies have pointed out that strong shear caused by ISWs poses a serious threat to marine engineering, submarines and acoustic communications^10,26,33–37^. Therefore, it is necessary to forecast ISWs in marine engineering or marine communication. It is particularly important to accurately and automatically identify ISWs (period, amplitude, start time and end time), which are significant for the forecast of ISWs. Scholars usually do not pay too much attention to the identification of ISWs. There are various methods to identify ISWs^3,30,38^. For the usual academic studies of ISWs, the identification of ISWs is not a problem. It does not require automatic and accurate identification of ISWs. Scholars can use different methods to identify ISWs. If necessary, scholars can manually supplement the missing ISWs later, or only need to identify a part of ISWs. There is no doubt that in these studies, the present methods for identifying ISWs are fully capable of meeting their research needs, and the methods are simple and effective. However, there are some limitations in applying the present methods of identifying ISWs to marine engineering. The present identification methods usually identify ISWs by calculating the amplitude of each ISW. However, the amplitudes of ISWs calculated by the identification methods themselves are not accurate, so it is difficult to automatically identify all ISWs. In marine engineering, missing the forecast of ISWs or misjudging their energies will cause serious consequences. Therefore, a new method can avoid calculating amplitude to some extent, and can automatically and accurately identify the ISWs from the real-time observation and transmission of upstream data, is necessary, and this is the motivation of this study.

The frequent occurrence of ISWs in the SCS provides a good opportunity for us to study them. We deployed a mooring system in the northern SCS to measure the temperature covering the water depth, and obtained 130-day continuous time series. Many ISWs were found at the mooring station during the observation period. In this study, we first compared the traditional methods of identifying ISWs. On this basis, we proposed an improved method, which could identify ISWs more comprehensively and efficiently. This paper is organized as follows. “Results” section show and analyze the processes and results of ISWs identified by different methods. “Discussion” section analyzes the reasons for the difference in the number of ISWs identified by different methods.

## Data and methods

**Figure 1:**
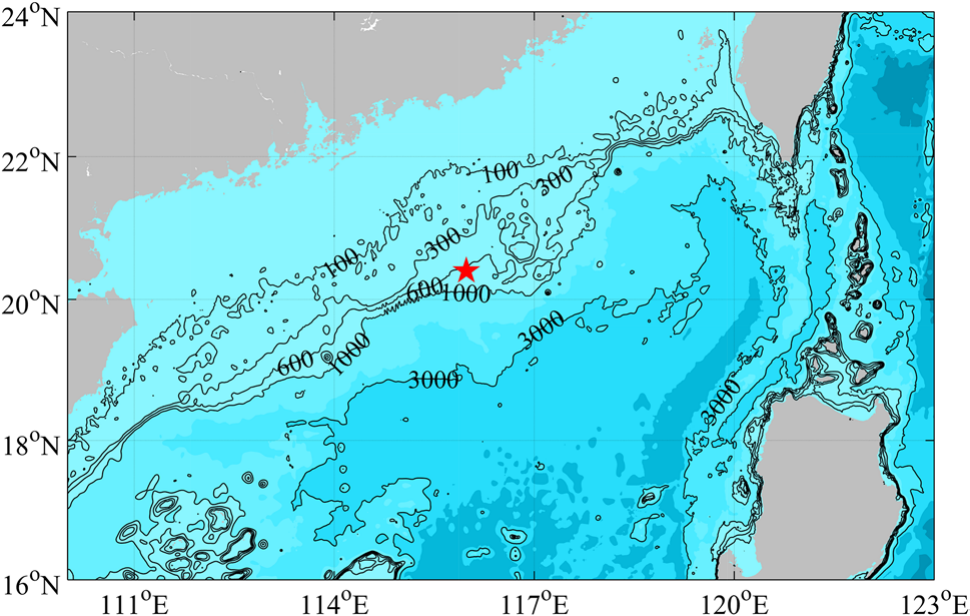
Map of the mooring near the Dongsha Islands in the SCS. The red pentagram denotes the mooring station. The solid lines and shadow denote the water depth. Figures are plotted using MATLAB R2016b (http://www.mathworks.com/).

From 22 July to 20 December 2018, a mooring system was deployed on the continental slope (115.55° E, 20.68° N) on the west side of the Dongsha Islands in the northern SCS to measure temperature. The water depth of the observation station is 662 m. The temperature profile and depth were measured by conductivity-temperature-depth (CTD) at every 50 m and by temperature loggers at every 10 m. Note that CTDs measured both temperature and depth, while temperature loggers only measured temperature. The sampling intervals of CTDs and temperature loggers are 30 s. The mooring station is shown in Fig. 1.

### Temperature data

The raw temperature data need to be preprocessed, including outlier elimination, depth correction and data standardization. During the observation period, there are a few outliers due to the influence of the external environment or the failure of the instrument itself, and these outliers need to be eliminated. Hence, the obvious outliers in the temperature data measured by the temperature loggers and the CTDs were eliminated. Because the temperature data measured by temperature loggers have no corresponding depth, we need to obtain the depth through depth correction. According to the positions of temperature loggers and CTDs installed on the mooring, the depths of temperature loggers between two adjacent CTDs were obtained by inwardly linear interpolation using the measured depth of CTDs. Finally, the temperature data were interpolated to the standard depth layers with uniform 5 m vertical intervals.

### World ocean atlas dataset

**Figure 2:**
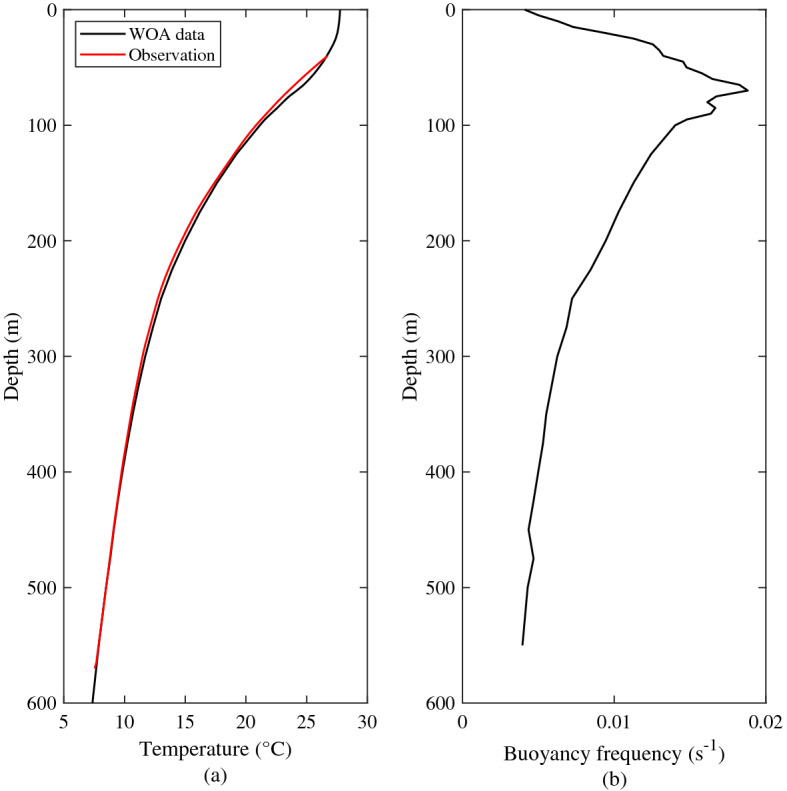
Vertical profiles of stratification. (**a**) Vertical profile of temperature from the WOA13 dataset (black curve) and measured temperature data (red curve). (**b**) Vertical profile of buoyancy frequency calculated with the WOA13 dataset.

The World Ocean Atlas 2013 (WOA13) is a gridded dataset based on actual observations, with a maximum spatial resolution of 0.25°. It provides the average values of temperature, salinity and other elements on a monthly, seasonal and annual basis at 102 standard depths ranging from 0 to 5500 m. Due to the lack of observations in the upper layer, the WOA13 data were used as a supplement. Figure 2a compares the average temperature from observations and monthly WOA13 data. As shown, the WOA13 data show a good agreement with observations. Hence, the WOA13 data were used to calculate the buoyancy frequency (*N*, Fig. 2b). According to the buoyancy frequency at the mooring station, the maximum buoyancy frequency (*N* > 0.017 s^−1^) appears at 55–90 m depth, which corresponds to location of the strongest thermocline.

### Ensemble empirical mode decomposition method

The ensemble empirical mode decomposition (EEMD) is a method of signal decomposition^39^, which overcomes the phenomenon of empirical mode decomposition (EMD) mode aliasing. The EEMD method decomposes the signal according to the time scale characteristics of the data, without setting the basis function in advance. Therefore, it can be applied to the decomposition of any signal in theory, and has a high signal-to-noise ratio. The core of the EEMD method is to extract the intrinsic mode function (IMF)^39,40^.

### Identification of ISWs

Generally, ISWs can be identified and extracted from temperature, density or current velocity data. Chen et al. used the depth displacement of isotherms from 11 to 28 °C to quantify the fluctuation characteristics of the isotherms by extracting the maximum and minimum values^30^. The depth distance and time between the adjacent maximum and minimum values are regarded as the amplitude and half of period of the ISW, respectively. According to the characteristics of ISWs in the northern SCS, the amplitudes of ISWs are usually greater than 10 m, and the periods are generally between 9 min and 2 h. Here, we define this method as whole water column isotherm (WCI) method.

Cui et al. used the depth displacement change of the 17 °C isotherm to determine ISWs^38^. If the depth variation of the isotherm exceeds 20 m within 30 min, it is determined as an ISW, and the depth variation is defined as its amplitude. Zhao and Alford extracted the 21 °C isotherm displacement from the original temperature profile, and used a bandpass filter to decompose the 21 °C isotherm displacement into three frequency bands^3^: subtidal component, tidal component and high frequency components. The high frequency component is composed of large amplitude ISWs and small amplitude internal waves. Here, we define this method as single isotherm (SI) method.

**Figure 3:**
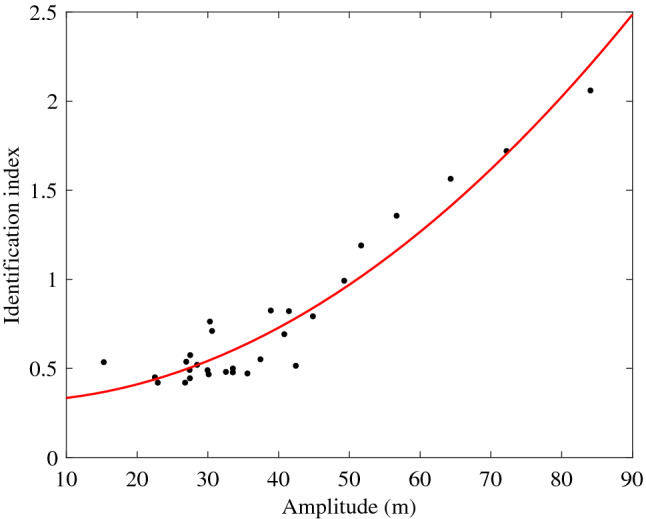
The variation of identification index with amplitudes of ISWs. The coordinates of x-axis of black points are the amplitudes *A*_*m*_ of ISWs, while the coordinates of y-axis are the values corresponding to the ISWs in the *T*_*em*_. The red curve is the least-squares fitted curve of the black points.

This paper proposes a new temperature superposition (TS) method to identify ISWs. Because ISWs can affect the whole water column, and the unique single-peak structure of the ISWs leads to the regular changes of thermodynamic characteristics of seawater from the seafloor to sea surface. For mode-1 ISWs, superimposing the temperatures at all depths will amplify the temperature fluctuations caused by the ISWs, and there will be a peak that is obviously different from the surroundings. Therefore, mode-1 ISWs can be identified by setting an identification index to find peaks. The temperature changes caused by mode-2 ISWs are opposite in the upper and lower layers, they are usually dominated by one of them. Taking the concave mode-2 ISWs as an example, when the temperatures at all depths are superimposed, the result is often dominated by the rise of temperature. The temperatures of all depths are superimposed and an identification index is set to find the peak. At the same time, the isotherms of temperature decrease are flipped up and down, then the temperatures at all depths are superimposed, and the second index is set to find the peak. The ISW is identified when two identification indexes find the same peak. Therefore, this method can be also used to identify and extract the mode-2 ISWs. In this paper, we mainly identify the mode-1 ISWs when their amplitude is greater than 20 m. The processes of identifying ISWs are displayed as follows:

1. The measured temperature *T*_*m*_ is filtered into filtered sequence *T*_*f*_ by a bandpass filter with a cut-off period of 5 min and 2 h. It is worth noting that the 5 min and 2 h are specified values according to the characteristics of ISWs in the northern SCS. These values can be changed in different areas and under different conditions. At the same time, the measured temperature *T*_*m*_ is decomposed into *T*_*e*_ by EEMD method.
2. Both *T*_*f*_ and the signals of the first five IMFs of *T*_*e*_ are depth averaged to obtain two new temperature time series *T*_*fm*_ and *T*_*em*_ respectively. The reason for selecting the first five IMFs is that ISWs are mainly included in the first five IMFs. For a detailed analysis, see the Temperature Superposition Method section below.
3. Figure 3 shows the variation of identification index with amplitudes of ISWs. The identification indexes are different for temperature data with different water depths and different vertical layers, In the *T*_*fm*_ or *T*_*em*_ sequence, by finding the values at the moment when the amplitudes *A*_*m*_ of ISWs are the maximum, the sequence *I*_*ds*_ corresponding to different amplitudes of ISWs can be obtained. *A*_*m*_ and *I*_*ds*_ are fitted by the least square method to obtain the identification index curve that varies with amplitude (Fig. 3). For example, this paper aims to identify the ISWs with amplitudes exceeding 20 m, and the identification index is 0.42. Thereafter, extract the extreme values of *T*_*fm*_ and *T*_*em*_, and identify the ISWs by selecting the extreme values that exceed the identification index. The time corresponding to the extreme value is the moment when the amplitude of the ISW is the largest. The start and end time of ISW are the time when the first extreme point closest to 0 is found forward and backward from the extreme point. The amplitude of ISW is the maximum depth displacement of all isotherms in the whole water column during the period of the ISW.

## Results

### ISWs identified by SI

**Figure 4:**
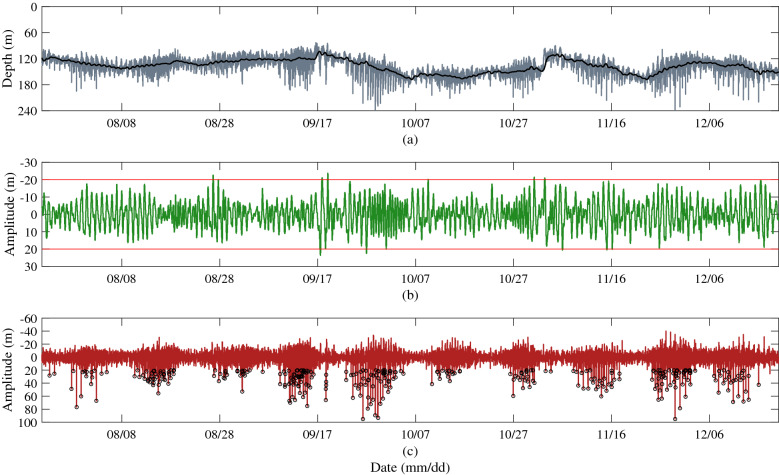
Schematic of identification of ISWs by SI method. (**a**) The 18 °C isotherm displacements (black) and the subtidal components (gray). (**b**) The internal tide components. The red lines are 20 m amplitudes. (**c**) The high frequency components. The downward spike-like fluctuations (small black circles) are the large amplitude ISWs. The frequency limits of (**a–c**) are 0.4 cpd, 0.4–2.5 cpd and 2.5 cpd, respectively.

According to the method of Zhao and Alford^3^, the isotherm displacement of 18 °C is selected to identify ISWs in this paper. Figure 4 shows the ISWs identified according to the SI method. A total of 343 ISWs with amplitude exceeding 20 m are identified by this method. Figure 4a shows the depth displacements of the 18 °C isotherm extracted from the original temperature profile and the subtidal component extracted using a low-pass filter with cut-off frequency of 0.4 cycles per day (cpd). Figure 4b shows the internal tidal component obtained using a bandpass filter with cut-off frequency of 0.4–2.5 cpd. Figure 4c is the high frequency component (the cut-off frequency is 2.5 cpd), which are composed of large amplitude ISWs and small amplitude internal waves. It can be seen that the depths of the subtidal component corresponding to the 18 °C isotherm at the mooring station are mainly between 103.26 and 168.32 m, and the maximum depth displacement is 65.06 m. The amplitudes of tidal component are mainly less than 20 m, and the maximum depth displacement is 47.38 m. The maximum depth displacement of high frequency internal waves can reach 135.17 m, and the ISWs are included in these high frequency internal waves.

### ISWs identified by WCI

**Figure 5:**
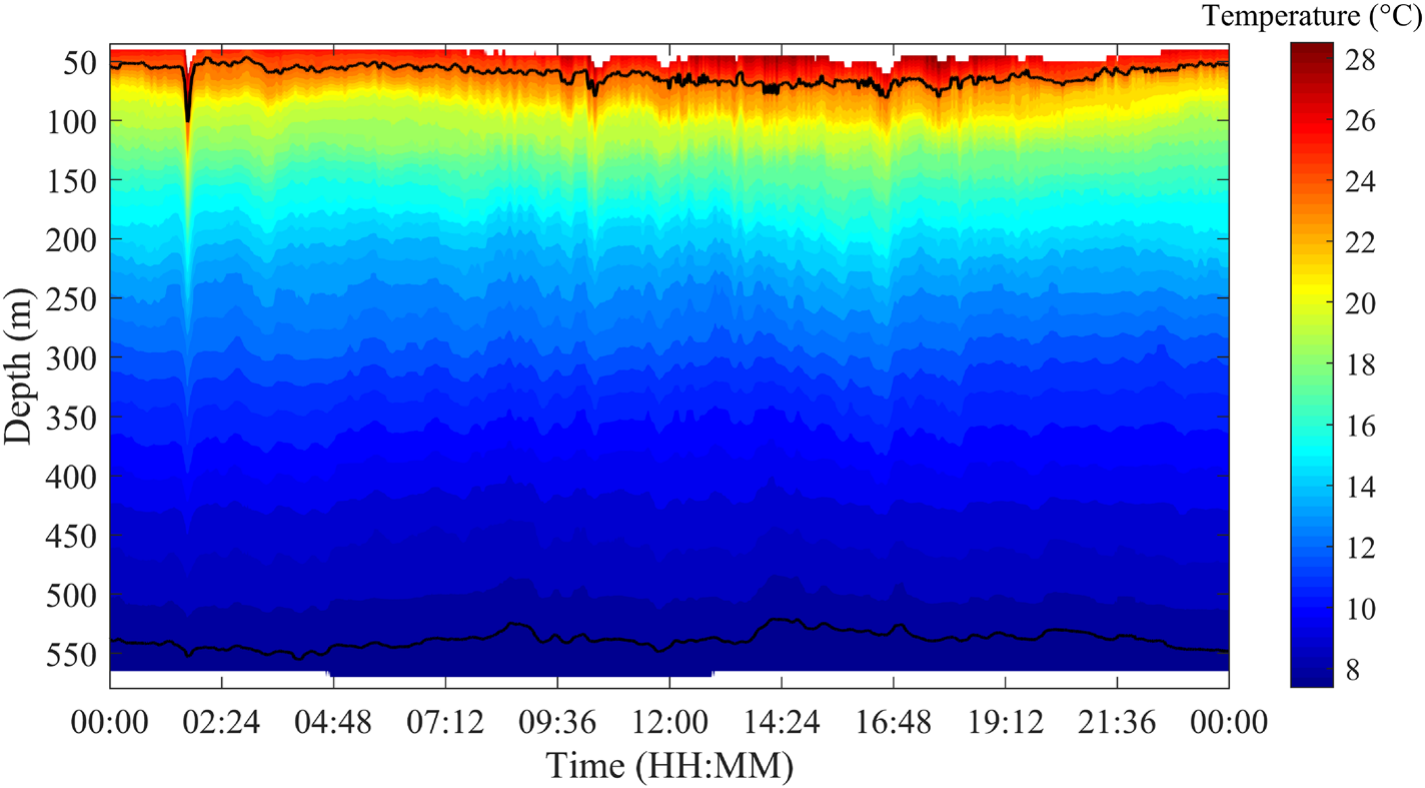
Spatio-temporal distribution of temperature (shadow) on July 29, 2018. The black lines denote 8 and 24 °C isotherms, respectively.

**Figure 6:**
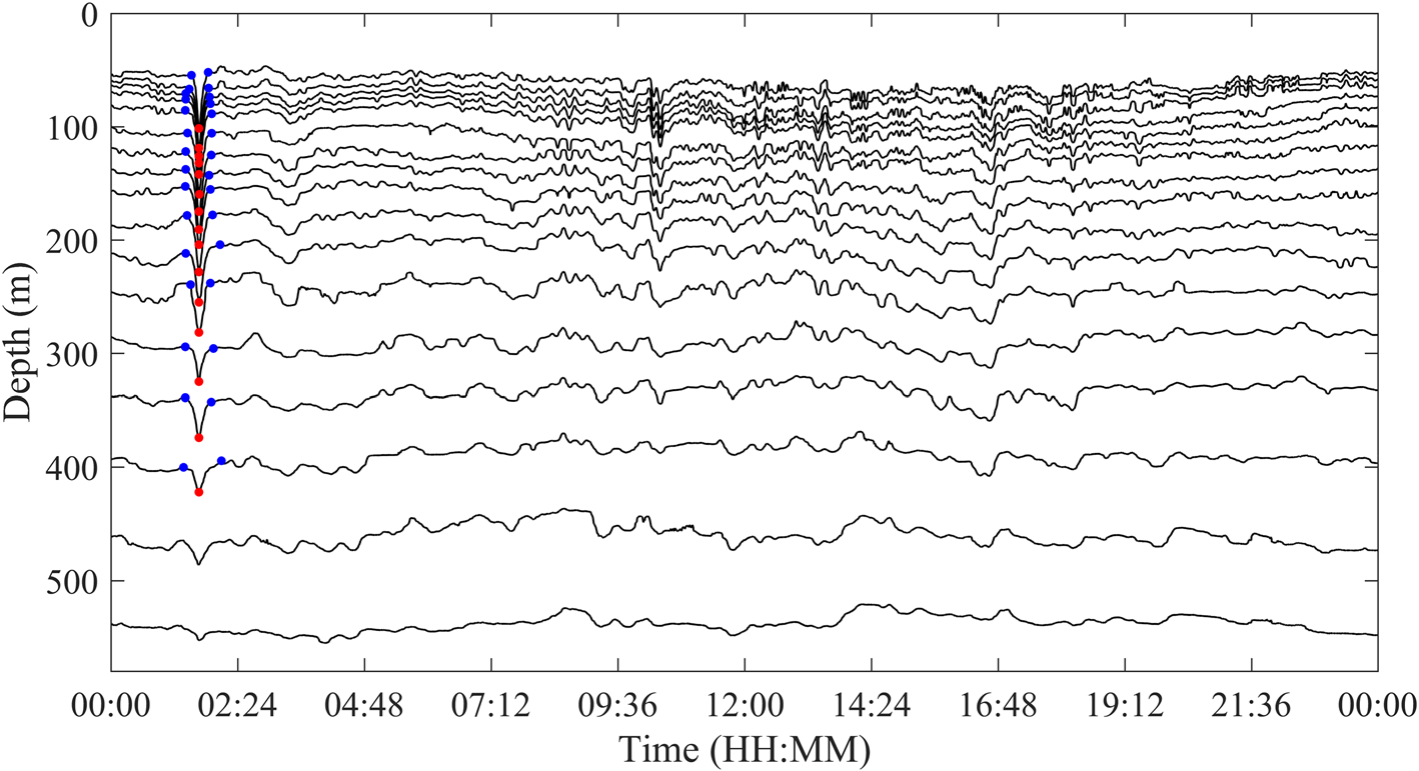
Schematic of identification of ISWs by WCI method on July 29, 2018. The black lines are the isotherms from 8 to 24 °C, the red dots correspond to the time when the amplitude of the ISW is the largest, and the blue dots to the left and right of the red dots are the start and end time of the ISW, respectively.

In this paper, the depth displacements of the 8–24 °C isotherms are selected to identify ISWs. Figure 5 shows time-depth distribution of temperature on July 29, 2018. It can be clearly seen that there are ISWs between 0:00–2:00, 9:36–12:00 and around 16:00. The amplitude of the first ISW is the largest, and the amplitudes of the latter two ISWs are smaller. Figure 6 shows a schematic of the ISWs identified by whole water column isotherm (WCI) method, the depth displacements of isotherms meeting the identification conditions are marked with red and blues dots. The red dots correspond to the time when the amplitude of the ISW is the largest, and the blue dots to the left and right of the red dots are the start and end time of the ISW, respectively. A total of 380 ISWs with the amplitude exceeding 20 m are identified.

### ISWs identified by TS

**Figure 7:**
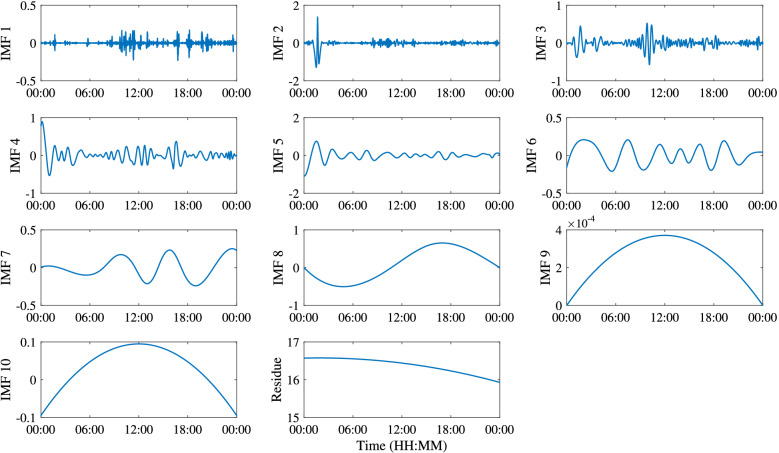
The IMFs and residue of EEMD of temperature at a depth of 155 m on July 29, 2018.

**Figure 8:**
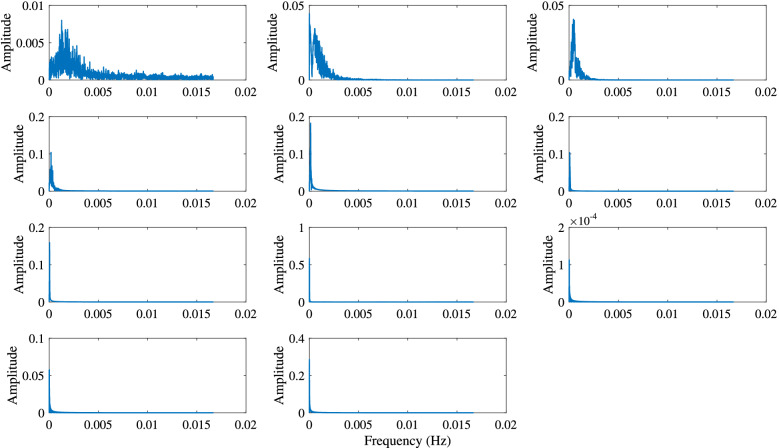
Frequencies of IMFs of EEMD of temperature at a depth of 155 m on July 29, 2018.

**Figure 9:**
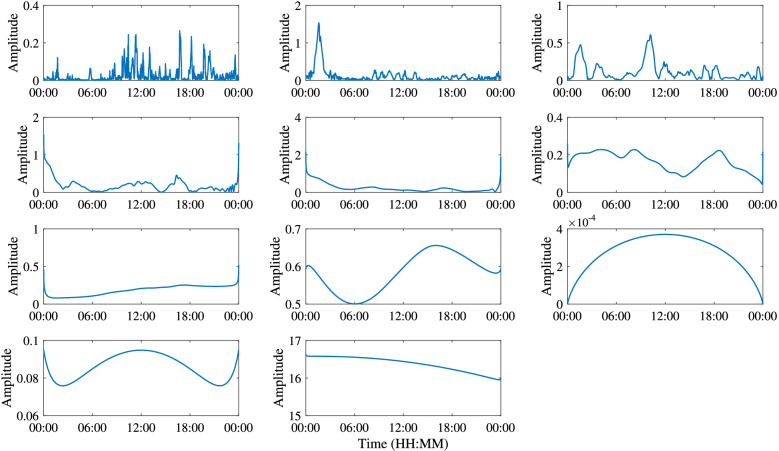
Instantaneous amplitude of IMFs of EEMD of temperature at a depth of 155 m on July 29, 2018.

**Figure 10:**
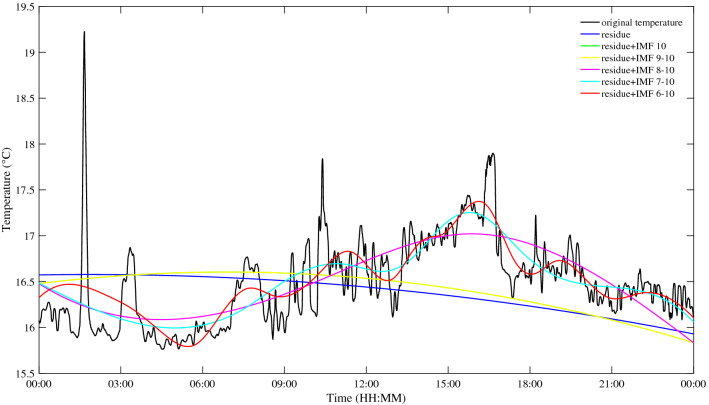
Superposition of different IMFs and residue of EEMD of temperature at a depth of 155 m on July 29, 2018.

The commonality of ISWs is analyzed by decomposing the temperature at different depths by the EEMD method. Taking the temperature at 155 m depth as an example, Fig. 7 shows the IMFs and the residue after EEMD on July 29, 2018. Each IMF component contains the local characteristic information of different scales of the original temperature signal. By EEMD, the temperature signal at 155 m is decomposed into 10 IMFs and a residue. Figure 8 shows the frequency of each IMF component decomposed by EEMD. The first five IMFs contain signal periods below 3 h, and from the sixth IMF, the corresponding signal periods are basically greater than 3 h. The peak frequencies of IMF 1–6 are 0.0013 Hz, 5.45 × 10^–4^ Hz, 4.39 × 10^–4^ Hz, 1.87 × 10^–4^ Hz, 1.30 × 10^–4^ Hz and 6.51 × 10^–5^ Hz, corresponding to the main signal periods of 13.13 min, 30.57 min, 37.93 min, 89.04 min, 128.13 min and 256.41 min, respectively. Similarly, data measured on other dates also show the same results, which are not presented here. Results show that in the northern SCS, the ISWs are mainly in the first five IMFs. From IMF 6, the signals are mainly tidal and subtidal signals. Figure 9 shows the instantaneous amplitudes of the IMF components decomposed by EEMD. There are obvious signals with larger amplitudes in the first five IMFs. According to the above identification of the ISWs in Fig. 5, the ISWs with larger amplitude occur at 01:40, 10:24 and 16:39, respectively. The comparison shows that the ISWs in IMF 2 and IMF 3 were relatively obvious, especially in IMF 3. For further analysis, we compare the temperature signal with the IMFs of EEMD at a depth of 155 m, and analyze the superposition of the latter five IMFs and residue and the comparison of the original temperature signals (Fig. 10). The results also show that the latter five IMFs and residue mainly include tidal and subtidal signals.

**Figure 11:**
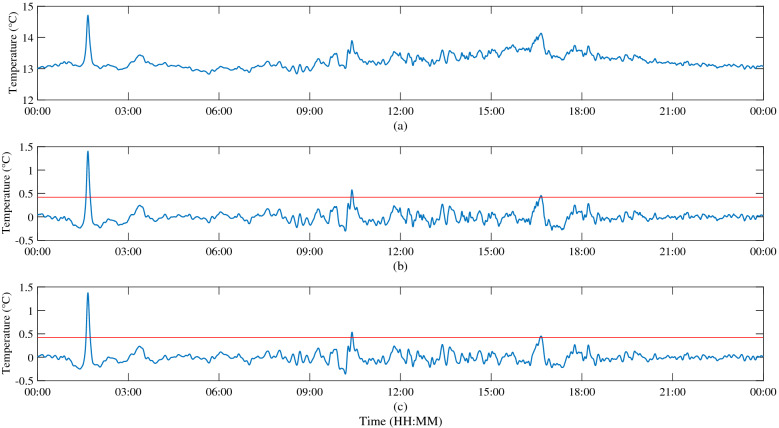
Schematic of identification of ISWs by TS method. (**a**) The depth-averaged temperature in the whole water column. (**b**) Sum of the first five IMFs of EEMD. (**c**) Sum of filtered depth-averaged temperature, the red lines are the index lines with a value of 0.42.

Figure 11 shows the ISWs identified by the temperature superposition (TS) method. A total of 415 ISWs with amplitude exceeding 20 m are identified by this method. Figure 11a shows the depth-averaged temperature obtained by averaging the original temperature over the depth. The ISW is a fluctuation that can affect the whole water column, the superposition of temperature over the whole depth will undoubtedly amplify the signal of the ISW. At the same time, some disturbing or small-amplitude internal waves will also be amplified. Therefore, it is necessary to provide an identification index to identify large amplitude ISWs. Figure 11b shows the sum of the first five IMFs obtained by the EEMD method. Compare with Fig. 11c, the superimposed temperature is averaged and the identification index is obtained. It can be seen that the signal of the ISW mainly distributes in the first five IMFs of EEMD.

## Discussion

The three methods used to identify ISWs are compared in this section. For comparability, we use the same method to recalculate the amplitudes of ISWs identified by three methods respectively. The TS method identifies the most ISWs, followed by WCI method, and the SI method identifies the fewest. Since the three methods have amplitude constraints when identifying ISWs, the sensitivity of these three methods to amplitude is shown in Table 1. The amplitudes of most ISWs occurred in the northern SCS from July to December are between 20 and 29 m. With the increase of amplitude, the number of ISWs decreased. During the observation period, the measured maximum amplitude of ISWs exceeded 100 m. In the range of 20–59 m, the SI method identifies the fewest ISWs (289), followed by the WCI method (326), and the TS method identifies the most (361). For ISWs with amplitudes larger than 60 m, all the three methods identify 54 ISWs. This result suggests that the three methods can well identify and extract ISWs with large amplitudes. For the SI method, a single isotherm cannot accurately reflect the amplitudes of all ISWs. For example, some ISWs meet the identification conditions, but their maximum amplitudes do not appear on the isotherm selected by the SI method. For the WCI method, it is more efficient to identify ISWs, as most of the temperature signals in the whole water column are considered. However, this method requires the ISWs to satisfy the identification conditions at more than 6 isotherms. In other words, the WCI method may miss the ISWs which meet the identification conditions at less than 6 isotherms. In contrast, the TS method takes the temperature signals of all depth layers into consideration, eliminates the influence of internal tidal signals, and amplifies the signals of ISWs to a certain extent, which leads to more accurate identification of ISWs.

**Table 1:** Number distribution of ISWs identified by SI, WCI and TS methods in different amplitude ranges.

Amplitude (m)	20–29	30–39	40–49	50–59	60–69	70–79	80–89	90–99	100–109	110–119
SI	100	88	55	46	27	16	6	2	2	1
WCI	106	110	61	49	27	16	6	2	2	1
TS	112	117	73	59	27	16	6	2	2	1

**Figure 12:**
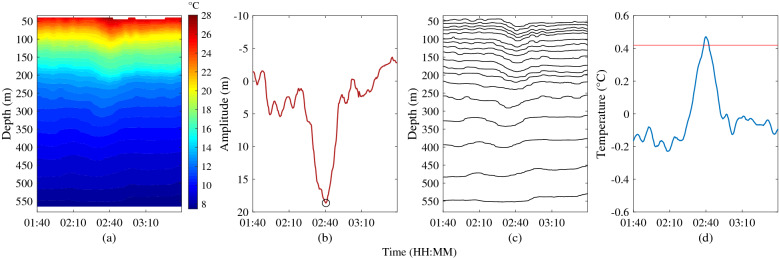
A case of identifying an ISW with an amplitude more than 20 m. (**a**) Spatio-temporal distribution of temperature on August 26, 2018. (**b**) ISW identified by SI method, the downward spike-like fluctuation (small black circle) is same as Fig. 4. (**c**) ISW identified by WCI method. (**d**) ISW identified by TS method, the red line is the identification index with a value of 0.42 corresponding to 20 m amplitude.

To compare the differences of the three methods more clearly, an ISW case is illustrated in Fig. 12. Figure 12a shows the spatio-temporal distribution of the temperature of an ISW on August 26, 2018. We can clearly see the ISW, whose amplitude is 28.48 m. Result of the SI method shows that the ISW exists, but its amplitude is underestimated to be less than 20 m, so the ISW cannot be identified by the SI method. When using the WCI method, we find that there is no isotherm that meet the identification conditions. This indicates that the maximum amplitude of this ISW does not occur on the selected isotherms. In other words, the ISW cannot be identified by the WCI method. For the TS method, the identification index corresponding to 20 m amplitude is 0.42. Hence, the ISW can be easily identified by the TS method. The SI and WCI methods are not absolutely unable to identify the ISW, but if the amplitude of identified ISWs is set to be larger than 20 m, the SI and WCI methods would miss this ISW.

## Conclusions

The accurate identification of ISWs is of great significance to ocean models and the forecast of ISWs. From July 22 to December 20, 2018, a mooring system was deployed in the northern SCS. The deployed CTDs and temperature loggers measured temperature from 50 to 580 m. The measured temperature data are used to study the methods of identifying and extracting ISWs. ISWs are fluctuations that can affect the whole water column, the superposition of temperature over the whole depth will undoubtedly amplify the signals of the ISWs. At the same time, some disturbing or small-amplitude internal waves will also be amplified. Therefore, it is necessary to provide an identification index to identify large amplitude ISWs. Based on this, we propose a new method to identify ISWs: the TS method. These data support the following conclusions.

In total, the TS method identifies the most ISWs, followed by the WCI method, and the SI method identifies the fewest ISWs. When the amplitudes of ISWs are smaller than 60 m, the number of ISWs identified by TS is the most. When the amplitudes of ISWs are greater than 60 m, the three methods can identify the same number of ISWs.

In the process of identifying ISWs, for the SI method, a single isotherm cannot accurately indicate the maximum amplitudes of all ISWs, and these ISWs are not be identified by the SI method. For the WCI method, it requires the ISWs to satisfy the identification conditions at more than 6 isotherms. In other words, the WCI method may miss the ISWs which meet the identification conditions at less than 6 isotherms. The TS method takes the temperature signals of all depth layers into consideration, eliminates the influence of internal tidal signals, and amplifies the signals of ISWs to a certain extent, which leads to more accurate identification of ISWs.

## Data Availability

The datasets generated and analyzed during the current study are not publicly available due the requirements of the investigative project, but are available from the corresponding author on reasonable request.
